# High-Throughput Computing to Detect Harmful Drug-Drug Interactions in Older Adults: Protocol for a Population-Based Cohort Study

**DOI:** 10.2196/77224

**Published:** 2025-10-10

**Authors:** Neda Rostamzadeh, Rishabh Sharma, Sheikh S Abdullah, Eric McArthur, Niaz Chalabianloo, Jessica M Sontrop, Matthew A Weir, Kamran Sedig, Amit X Garg, Flory T Muanda

**Affiliations:** 1 Department of Computer Science Western University London, ON Canada; 2 London Health Sciences Centre Research Institute London, ON Canada; 3 ICES Ontario London, ON Canada; 4 Department of Computer Science MacEwan University Edmonton, AB Canada; 5 Department of Physiology and Pharmacology Western University London, ON Canada; 6 Department of Epidemiology and Biostatistics Schulich School of Medicine and Dentistry Western University London, ON Canada; 7 Division of Nephrology, Department of Medicine Schulich School of Medicine and Dentistry Western University London, ON Canada

**Keywords:** older adults, high-throughput computing, drug-drug interactions, machine learning, drug safety

## Abstract

**Background:**

Drug-drug interactions (DDIs) are a major concern, especially for older adults taking multiple medications. Although Health Canada and the US Food and Drug Administration (FDA) use population-based studies to identify adverse drug events, detecting harmful DDIs is challenging due to the millions of potential drug combinations. Traditional pharmacoepidemiologic studies are slow and inefficient, often missing important harmful DDIs.

**Objective:**

This protocol outlines a novel approach to efficiently identify harmful DDIs using administrative health care data.

**Methods:**

Using high-throughput computing, we will conduct multiple population-based, new-user cohort studies using Ontario’s linked administrative health care data. The cohorts will be selected from the population of Ontario residents aged 66 years and older who filled at least one oral outpatient drug prescription from 2002 to 2023. In each cohort, the exposed group will comprise individuals who are regular users of one drug (drug A) who start a new prescription for a second drug (drug B); the referent group will comprise regular users of drug A not taking drug B. We will evaluate 74 acute outcomes within 30 days of cohort entry, including hospitalizations, emergency department visits, and mortality. Propensity score methods will balance exposed and referent groups on more than 400 baseline health characteristics. Modified Poisson and binomial regression models will estimate risk ratios (RRs) and risk differences (RDs). To ensure findings are both statistically and clinically meaningful, we will apply prespecified thresholds for effect sizes (eg, lower bounds of 95% CIs≥1.33 for RRs and ≥0.1% for RDs) and control the false discovery rate at 5% using the Benjamini-Hochberg procedure to address multiplicity. Subgroup and sensitivity analyses, including negative control outcomes and E-values, will assess robustness.

**Results:**

In a preliminary analysis, we identified approximately 3.8 million older adults who filled prescriptions for over 500 unique medications during the study period (2002-2023), and therefore, approximately 200,000 potential drug combinations will be available for study. The initial drug pair cohorts had a median of 583 new users per cohort (IQR 237-2130); the median overlap in drug pair prescriptions was 57 days (IQR 30-90). The protocol was finalized on August 30, 2025, and outlines the analysis of data from 2002 to 2023. The analysis is scheduled to be completed by fall 2026, with results interpreted in 2027. The final manuscript submission is planned for December 2028.

**Conclusions:**

This study aims to identify credible signals of harmful DDIs in older adults in routine care. This study will use an innovative approach that leverages data from provincial administrative health care databases and integrates high-throughput computing and rigorous pharmacoepidemiologic methods to generate robust real-world evidence that can inform safer prescribing practices and regulatory decision-making.

**International Registered Report Identifier (IRRID):**

DERR1-10.2196/77224

## Introduction

Drug-drug interactions (DDIs) occur when two or more medications interact in ways that alter their effectiveness or safety [1,2]. The drug being affected is termed the *object drug*, while the drug causing the interaction is known as the *precipitant drug* [3,4]. The precipitant drug can influence the object drug through pharmacokinetic (PK) mechanisms (affecting absorption, distribution, metabolism, or excretion) or pharmacodynamic (PD) mechanisms (altering the drug’s effects at its target site) [3]. An interaction may increase the effect of an object drug, leading to toxicity. Alternatively, an interaction may lessen the effect of an object drug, leading to reduced efficacy or treatment failure [5].

DDIs pose a significant risk for older adults due to polypharmacy and age-related changes in drug metabolism and elimination [6-8]. Approximately one in six hospitalizations among older adults is attributed to adverse drug events [9]. Additionally, older adults are often excluded from clinical trials, limiting our understanding of how DDIs affect this population. Drugs with a narrow therapeutic index are particularly concerning, as even minor interactions can have serious clinical consequences [10,11].

Although premarket clinical trials evaluate PK DDIs in healthy volunteers, many clinically significant DDIs are only identified after drug approval through case reports, small observational studies, and large-scale pharmacoepidemiologic research [12,13]. Traditional DDI detection methods often analyze one drug pair at a time, which can be slow and inefficient. These limitations are a concern worldwide: international efforts using electronic health records, claims data, or national prescription registries have made progress, but such approaches may not scale fast enough to keep pace with the thousands of new drug combinations entering the market each year, highlighting the need for rapid, large-scale, and systematic surveillance methods.

High-throughput computing and automated analytical pipelines have been applied in other contexts to rapidly screen for potential DDIs. For example, researchers in the United States and Europe have used these approaches with large electronic health care databases to identify clinically relevant DDI signals [14,15]. Bykov et al [14] validated a case-crossover–based method by successfully flagging known interactions in large US claims databases. Similarly, Leonard et al [15] demonstrated the ability of high-throughput pharmacoepidemiologic screening using a self-controlled case series design to confirm known DDI risks and identify novel potential interactions in real-world data. Although Bykov et al [14] and Leonard et al [15] have demonstrated the potential of high-throughput screening for DDIs in US health care databases using self-controlled designs, our study complements and extends these approaches by applying high-throughput computing to Ontario’s population-based, linked administrative health care data within a universal health care system using a population-based, new-user cohort design [16]. This will allow us to systematically evaluate over 200,000 medication combinations in older adults, integrating PK, PD, and epidemiologic evidence for robust, real-world signal detection at a scale and scope not previously achieved. By applying these methods in a Canadian context, we aim to identify potentially harmful DDIs that may warrant further validation, address key knowledge gaps, and generate findings relevant to both national and international health care systems. To the best of our knowledge, this represents the first application of such an approach in Canada, leveraging the strengths of a universal health care setting to further enhance the generalizability and clinical relevance of detected DDI signals. The results of this work have the potential to support safer prescribing practices, generate signals that could inform clinical decision-making once validated, and guide health care policy aimed at reducing adverse drug events in older adults.

This protocol outlines our plan to detect harmful DDIs more efficiently in routine care using high-throughput computing and automation. To do this, we will simultaneously conduct more than 200,000 population-based, new-user drug safety studies using 21 years of data from Ontario’s administrative health care databases (2002-2023). These databases contain encrypted data on all health care visits and hospitalizations of Ontario residents—all of whom have universal access to hospital care and physician services through a government-funded single-payer system, with Ontarians aged 65 years and older also receiving universal outpatient prescription drug coverage.

Following the application of inclusion and exclusion criteria, we will analyze a refined set of medication cohorts, each containing an exposed group (individuals prescribed a certain pair of medications) and a referent group (individuals prescribed a single medication). The exposed and referent groups will be statistically balanced on more than 400 baseline health characteristics. We will assess 74 clinically relevant outcomes and conduct extensive sensitivity and bias analyses to minimize false discoveries. Harmful DDIs that meet prespecified criteria will be further examined in additional and exploratory analyses. This study’s objective is to identify credible signals of harmful DDIs in older adults in routine care.

## Methods

### Study Design and Data Sources

Using high-throughput computing and automation, we will conduct over 200,000 population-based, new-user drug safety cohort studies using linked administrative health care databases in Ontario, Canada. The study period will span from July 1, 2002, to March 1, 2023. All study data will be obtained from Ontario’s linked administrative health care databases housed at the Institute for Clinical Evaluative Sciences (ICES) [17]. These databases contain encrypted, person-level data for Ontario residents, who have universal access to hospital care and physician services through a government-funded, single-payer system. A description of the databases is provided in Table 1.

**Table 1:** Description of study databases.

Database		Description
**National databases/registries**		
	Canadian Institute for Health Information’s Discharge Abstract Database (CIHI-DAD)	Contains diagnostic and procedural information for all hospitalizations, collected by each province
	Canadian Organ Replacement Register (CORR)	National registry that includes information on vital organ transplantation and dialysis activities
	National Ambulatory Care Reporting System (NACRS)	Contains information on hospital and community-based ambulatory care visits
**Ontario databases**		
	Ontario Mental Health Reporting System (OMHRS)	Contains adult inpatient mental health information
	Registered Persons Database (RPDB)	Contains information on patient demographics, including sex, birth dates, and death dates
	ICES^a^ Physician Database (IPDB)	Contains physician-related information, such as birth dates, sex, education, and specializations
	Ontario Drug Database	Contains claims for prescription drugs received under the ODB^b^ program
	Ontario Health Insurance Plan (OHIP)	Contains diagnostic information and health claims for inpatient and outpatient services
	Ontario Laboratories Information System (OLIS)	Contains laboratory test orders and results from hospitals, community labs, and public health labs

^a^ICES: Institute for Clinical Evaluative Sciences.

^b^ODB: Ontario Drug Benefit.

Residents aged 65 years and older receive universal outpatient prescription drug coverage through the Ontario Drug Benefit (ODB) program (while residents of all ages only receive universal prescription drug coverage for medications dispensed in emergency departments or during hospital admissions). Consequently, our study will leverage comprehensive outpatient prescription data for residents aged 65 years and older. We have previously used these databases to examine adverse drug events and health outcomes [16,18-21].

These databases are expected to be complete for all study variables, except for prescriber specialty (10%-20% missing), rural residence, and neighborhood income quintile (less than 2% missing). The only source of loss to follow-up is emigration from Ontario, which occurs at an estimated rate of 0.5% per year [22].

**Figure 1:**
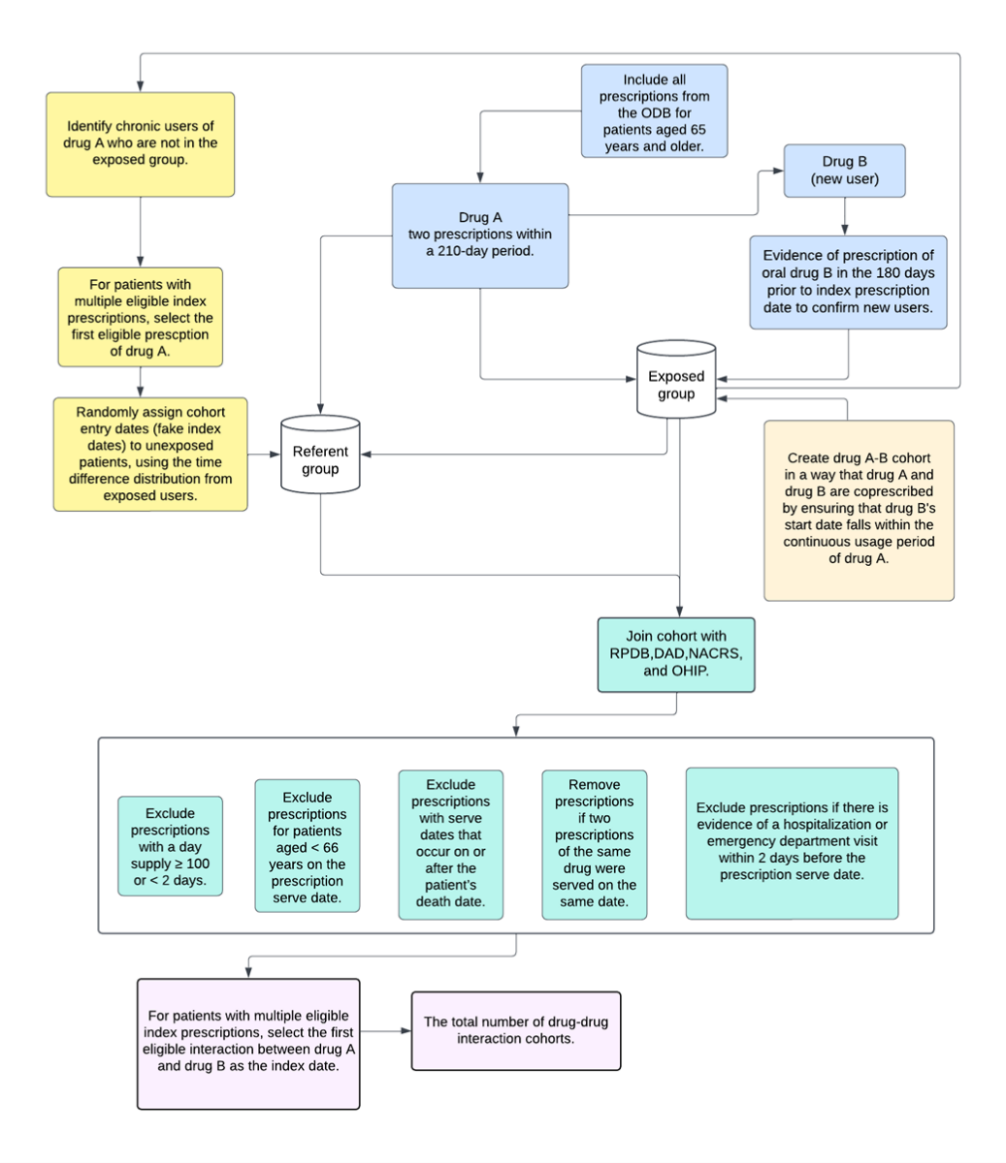
Study flow diagram. DAD: Discharge Abstract Database; NACRS: National Ambulatory Care Reporting System; ODB: Ontario Drug Benefit; OHIP: Ontario Health Insurance Plan; RPDB: Registered Persons Database.

The study conduct and reporting will adhere to recommended guidelines for observational pharmacoepidemiology studies that use routinely collected health data (Multimedia Appendix 1) [23,24]. More details on the databases, study variables, and the codes used to define baseline comorbidities are provided in Multimedia Appendix 2. The integration of data from different sources is summarized in Multimedia Appendix 3. The process of building the cohorts is summarized in Figure 1, and the analytic steps are summarized in Multimedia Appendix 4.

### Source Population

The source population will comprise adults aged 66 years and older who filled at least one oral outpatient drug prescription with a supply duration of at least 3 days, dispensed between July 1, 2002, and March 1, 2023 (codes for nonoral versions are provided in Multimedia Appendix 5). The age criterion will ensure that individuals in this population have at least 1 year of prior ODB prescription drug coverage.

### Cohort Builds

The study cohorts will be constructed using the process described later. Each cohort will be selected from the source population and contain an exposed group (individuals prescribed a pair of medications: drugs A and B) and a referent group (individuals prescribed drug A), as defined next.

#### Regular Users of Drug A

We will first select individuals with at least two prescriptions for the same medication (drug A) within a 210-day period, with a maximum allowable gap equivalent to 150% of the days’ supply of the previous prescription.

#### New Users of Drug B

From the sample of regular users of drug A, we will select individuals who start a prescription for a new medication (drug B) that is taken concurrently with drug A, meaning drug B’s start date is on the same date as the most recent drug A fill date or falls within drug A’s supply duration.

#### Cohort Entry Dates for Exposed and Referent Groups

The exposed group will comprise regular users of drug A and new users of drug B, as described earlier. The cohort entry date for individuals in the exposed group will be the start date of drug B. The referent group will comprise regular users of drug A who are not coprescribed drug B. The cohort entry date for individuals in the referent group will be assigned based on the distribution of the time differences between the most recent prescription for drug A and the initiation of drug B for individuals in the exposed group, ensuring similar observation periods between groups [25].

#### Individual-Level Exclusion Criteria Applied to the Exposed and Referent Groups

We will exclude individuals who meet any of the following criteria:

- Individuals prescribed drug B within 180 days before the cohort entry date (to ensure those in the exposed group are new users of drug B and that those in the referent group were not previously exposed).
- Individuals prescribed another medication in the same class as drugs A and B on the cohort entry date. For example, if drug A or B is sertraline, which is a serotonin reuptake inhibitor (SSRI), individuals prescribed a nonsertraline SSRI on the cohort entry date will be excluded.
- Individuals discharged from the hospital or emergency department within 2 days before the cohort entry date (to ensure that prescriptions are new outpatient prescriptions).
- Individuals whose day supplies for drug A or B are ≥100 days or <2 days.
- Individuals who are nonpermanent Ontario residents, are aged <66 or >105 years on the cohort entry date, or have a date of death on or before the cohort entry date (data cleaning exclusions).

An individual can only enter a cohort once; if an individual meets the eligibility criteria multiple times, the first eligibility date will serve as the cohort entry date.

#### Cohort-Level Exclusion Criteria

To ensure meaningful results, only cohorts with a minimum sample size of 1000 patients, with at least 200 individuals in the exposed group and 200 individuals in the referent group, will be considered for further analysis.

### Outcomes

We will examine 74 outcomes within 30 days of cohort entry (Textbox 1). The outcomes were chosen using a prespecified analytic approach and considering the clinical significance of the outcomes and the observed rates in the study region [16]. Many of the algorithms used to identify these outcomes have been validated in previous studies and demonstrate high sensitivity and specificity [26-30]. Shorter and longer follow-up times for outcomes will be examined in additional analysis (see the *Additional Analysis to Characterize the Signals* section).

**Final set of 74 clinically relevant outcomes (ordered alphabetically).**

**Clinically relevant individual outcomes (n=56):**

- Abdominal aortic aneurysm repair/aortic bypass
- Achilles tendon rupture
- All-cause mortality (prespecified)
- Arthroscopy
- Asthma
- Atrial fibrillation/flutter or other arrhythmia (pacemaker insertion, palpitations, tachycardia unspecified, atrioventricular block, supraventricular tachycardia, or other conduction disorders)
- Bowel obstruction
- Bradycardia
- Bronchoscopy
- Carotid endarterectomy
- Carotid ultrasound
- Cataract, unspecified
- Chest X-ray
- Cholecystitis, unspecified
- Clostridium difficile colitis
- Colposcopy
- Computed tomography: abdomen
- Computed tomography: head
- Computed tomography: thorax
- Cutaneous drug reaction
- Cystoscopy
- Depression
- Disorders of calcium metabolism
- Emergency department visit for any reason (prespecified)
- Gout
- Guillain-Barré syndrome
- Head trauma
- Hearing loss
- Hospital admission for any reason (prespecified)
- Hyperkalemia
- Hypoglycemia
- Hypokalemia
- Hyponatremia
- Intensive care unit admission
- Kidney stones
- Laparoscopy
- Liver disease toxicity
- Lower limb amputation
- Major abdominal surgery
- Major cardiovascular surgery
- Osteoarthritis
- Pancreatitis
- Parkinson’s disease
- Physician visit for any reason (prespecified)
- Pneumonia
- Renal biopsy
- Retinal detachment
- Rhabdomyolysis
- Rheumatoid arthritis
- Self-harm
- Sepsis
- Surgery (for any reason other than major abdominal surgery, major cardiovascular surgery, lower limb amputation and fracture, and major ortho procedure)
- Severe respiratory depression
- Urinary incontinence
- Urinary retention
- Venous thromboembolism

**Clinically relevant composite outcomes (n=18):**

- Acute dialysis or acute kidney injury
- Aplastic anemia (unspecified), neutropenia, or other agranulocytosis, or thrombocytopenia (unspecified)
- Cardiac arrest or ventricular arrhythmia
- Cardiac catheterization or coronary revascularization
- Colonoscopy or esophagogastroduodenoscopy
- Diabetic ketoacidosis, hyperglycemia, or hyperosmolar nonketotic coma
- Delirium or other encephalopathy
- Delirium, fall, or other encephalopathy
- Echocardiography or Holter monitoring
- Electroencephalography or seizures
- Emergency department visit or hospital admission for any reason (prespecified)
- Fall, fracture or major orthopedic procedure, or fracture
- Fall or hypotension
- Fall or syncope
- Heart failure or myocardial infarction (most-responsible diagnosis), or ischemic stroke (most-responsible diagnosis)
- Hemarthrosis or hemorrhage (not elsewhere classified), intracerebral hemorrhage (unspecified), lower gastrointestinal bleed, other nontraumatic intracranial hemorrhage, subarachnoid hemorrhage (unspecified), or upper gastrointestinal bleeding
- Hypotension or syncope
- Urinary tract infection or urine culture

### Statistical Analysis Plan

All analyses will be programmed and automated using Python, SAS, the JavaScript library D3, and R (R Foundation for Statistical Computing) and will be conducted on the Health Artificial Intelligence Data Analytic Platform, a high-performance computing environment for analyzing ICES data. The open source JavaScript library D3 will be used to develop interactive visualizations.

#### Analysis to Balance the Exposed and Referent Groups in Each Cohort

Propensity scores will be calculated using multivariable logistic regression, incorporating more than 400 baseline characteristics (the complete list and coding definitions are provided in Multimedia Appendix 2). Characteristics include calendar year, prior use of drug A, demographics (eg, age, sex, neighborhood income quintile, urban vs rural residence, geographic region [31], and long-term care home residency), comorbidities (eg, asthma, diabetes, coronary artery disease) assessed over the 5 years before cohort entry, health care use in the year prior (eg, hospitalizations, emergency room visits, specialist consultations), and prescription drug dispensing within 120 days before cohort entry. The 120-day lookback period accounts for potential delays in prescription refills, ensuring comprehensive capture of medication dispensing.

The propensity scores will estimate the probability of a patient being in the exposed group versus the referent group, given their baseline characteristics. To enhance the comparability of the exposed and referent groups, we will exclude (1) individuals in the referent group with propensity scores lower than the lowest 1% of scores in the exposed group and (2) individuals in the exposed group with propensity scores higher than the highest 99% of scores in the referent group. This will also ensure that we do not violate the positivity assumption in causal inference, which requires that there should be a nonnull probability across each stratum of baseline characteristics within both the exposed and referent groups [32].

Inverse probability of treatment weighting (IPTW) using average treatment-effect-in-the-treated (ATT) weights will then be used to balance the exposed and referent groups on baseline characteristics as follows: Individuals in the referent group will be weighted using ATT weights, defined as their odds of being in the exposed group (propensity score/[1 − propensity score]). Individuals in the exposed group (the medication pair group) will receive weights of 1. This method will produce a weighted pseudosample of individuals in the referent group with a distribution of measured characteristics similar to that of the exposed group [33,34]. Between-group differences in baseline characteristics in the weighted samples will be examined using standardized differences (SDs) [35].

Cohorts will be excluded from further analysis if fewer than 95% of the baseline characteristics are balanced between the exposed and referent groups (ie, the SDs must be <10% for ≥95% of the characteristics).

#### Regression Analyses

A visual summary of the regression and additional exploratory analyses is provided in Multimedia Appendix 4. In each cohort, outcomes will be eligible for analysis if there are at least six outcome events in both the exposed and referent groups. Reporting fewer than six events could lead to potential patient reidentification and substantial estimation errors.

For each eligible cohort, we will use a modified Poisson regression model [36] to estimate the weighted risk ratio (RR) and its 95% CI for each outcome in the exposed group compared to the referent group. Additionally, a binomial regression model with an identity link function will be used to estimate the weighted risk difference (RD) and its 95% CI. If all 74 outcomes for a particular cohort are eligible, up to 74 regression models will be run.

#### Criteria for Identifying Potential Signals of Harmful DDIs

Associations between a drug pair and an adverse outcome that meet the following criteria will be considered potential signals of harm and analyzed further:

- Thresholds for RRs and RDs. The lower bound of the 95% CI for the RR must be ≥1.33, and for the RD, it must be ≥0.1%. These thresholds were chosen to ensure that only clinically significant increases in risk (of sufficient magnitude to be of concern) are considered. This analysis will focus solely on potential harms from drug combinations rather than protective benefits.
- Statistical significance. The *P* values from the modified Poisson regression model (for RRs) and the binomial regression model (for RDs) must be statistically significant after applying the Benjamini-Hochberg correction. This correction accounts for multiple comparisons across all outcomes and drug pair cohorts. The false discovery rate will be set at 5%, and the highest *P* value below the Benjamini-Hochberg critical value will be considered statistically significant, along with all *P* values below it. This correction will minimize the risk of false-positive discoveries (type I errors).

Drug pair cohorts that do not generate significant signals will not undergo further analysis.

#### Sensitivity and Bias Analyses to Guard Against Spurious Discoveries

Signals of potentially harmful DDIs will undergo the following sensitivity and bias analyses to ensure that identified signals are not artifacts of statistical or methodological bias but are credible associations worthy of further investigation:

- Survival analysis. To assess whether the initial signal persists when analyzed using a time-to-event approach, we will conduct a Cox proportional hazards regression with a 30-day follow-up period, censoring on death when all-cause mortality is not the outcome of interest. We will test the proportional hazards assumption by including an interaction term between the new-user status and follow-up time. This analysis will help determine whether the signal is robust across different modeling techniques.
- Negative-control exposure analyses. To detect potential biases from unobserved confounders, we will perform negative-control exposure analyses. A negative-control exposure is a variable that shares the same sources of bias as the drug of interest but cannot plausibly cause the outcome. We will rerun the regression analyses for the initial signals using a control exposure period starting 90 days before cohort entry [37]. This analysis will help identify any confounding factors that may have influenced the initial signal. Notably, the outcome of all-cause mortality cannot be examined with this method, as all patients lived to their cohort entry date. A positive association (ie, RRs and RDs significantly different from null, with *P*<.05) in this analysis would suggest potential confounding, whereas the absence of an association would bolster the credibility of the original signal.
- E-value analysis. We will conduct E-value analyses to quantify the minimum strength of association an unmeasured confounder would need to have with both the drug combination and the outcome to negate the observed signal [38]. Larger E-values indicate that substantial unmeasured confounding would be required to explain away the signal, whereas smaller E-values suggest that minimal unmeasured confounding could do so. For an initial signal to be considered robust, the E-value must exceed 2.
- Propensity score matching. To further validate the robustness of the signal, we will use 1:1 propensity score matching to balance the exposed and referent groups on baseline characteristics [39]. This method estimates the average treatment effect in the treated and mitigates the influence of extreme weights, which can occur with IPTW. Consistency between the results of this analysis and the initial findings will strengthen the validity of the signal.
- Bootstrap analysis. We will use a multiplier bootstrap procedure to compute RRs and RDs across 200 random samples, each of the same size as the original cohort. This method helps correct SEs and CIs, ensuring they have the correct coverage rates [36,40]. For the signal to be considered robust, at least 90% of the bootstrap samples must yield results consistent with the original findings, with *P*<.05 and CIs for RRs and RDs in the same direction as initially observed.

#### Additional Analysis to Characterize the Signals

Signals of potentially harmful DDIs that are supported by the aforementioned sensitivity and bias analyses will be further explored and characterized as follows:

- Longer and shorter follow-up periods. To better understand the effects of prolonged medication exposure, we will extend the follow-up period beyond 30 days. We will rerun the regression analyses to examine the RRs and RDs for outcomes occurring within 60 and 90 days after cohort entry. We will also assess shorter follow-up periods by examining outcomes that occur within 7 and 14 days of cohort entry.
- Number needed to harm (NNH). To further quantify the absolute risk, we will calculate the NNH, which is derived from the RD (NNH=1/RD). This metric indicates how many patients would need to take the medication combination to result in harm to one additional patient who otherwise would not have been harmed. A lower NNH indicates a greater potential for harm [41].
- Population attributable fraction (PAF). We will calculate the PAF using the formula Pe(relative risk − 1)/[1 + Pe(relative risk − 1)], where Pe represents the proportion of the population exposed to the drug combination [42]. A PAF close to 1 suggests that eliminating the drug pair exposure could significantly reduce the number of adverse outcomes in the population, while a PAF close to 0 implies that reducing exposure would have minimal impact at the population level. Separate PAFs will be calculated for each outcome, and the results will be combined as a composite outcome before calculating the PAF for each cohort.

#### Bidirectional Exposure Analysis to Assess Signal Robustness

We will specifically highlight instances where the addition of drug B to drug A, and, conversely, drug A to drug B, yield concordant results for the outcome of interest. Observing consistent effects in both directions, particularly for chronic medications, will strengthen confidence in the validity of the signal and suggest that findings are less likely to be explained by residual confounding or treatment selection bias.

#### Exploring the Data Using Interactive Visualization

To facilitate data exploration, we will develop an interactive visualization tool. The tool will enable users to visually explore the outputs from our analyses, providing an overview of the data with the ability to access, filter, and modify the displayed information [43,44]. Users will be able to apply filters based on RRs or other metrics to identify associations that meet specific criteria. The tool will support manual exploration, enabling users to interactively query and sort the data.

#### Overall Framework to Identify, Characterize, and Validate Signals of Harmful DDIs

**Figure 2:**
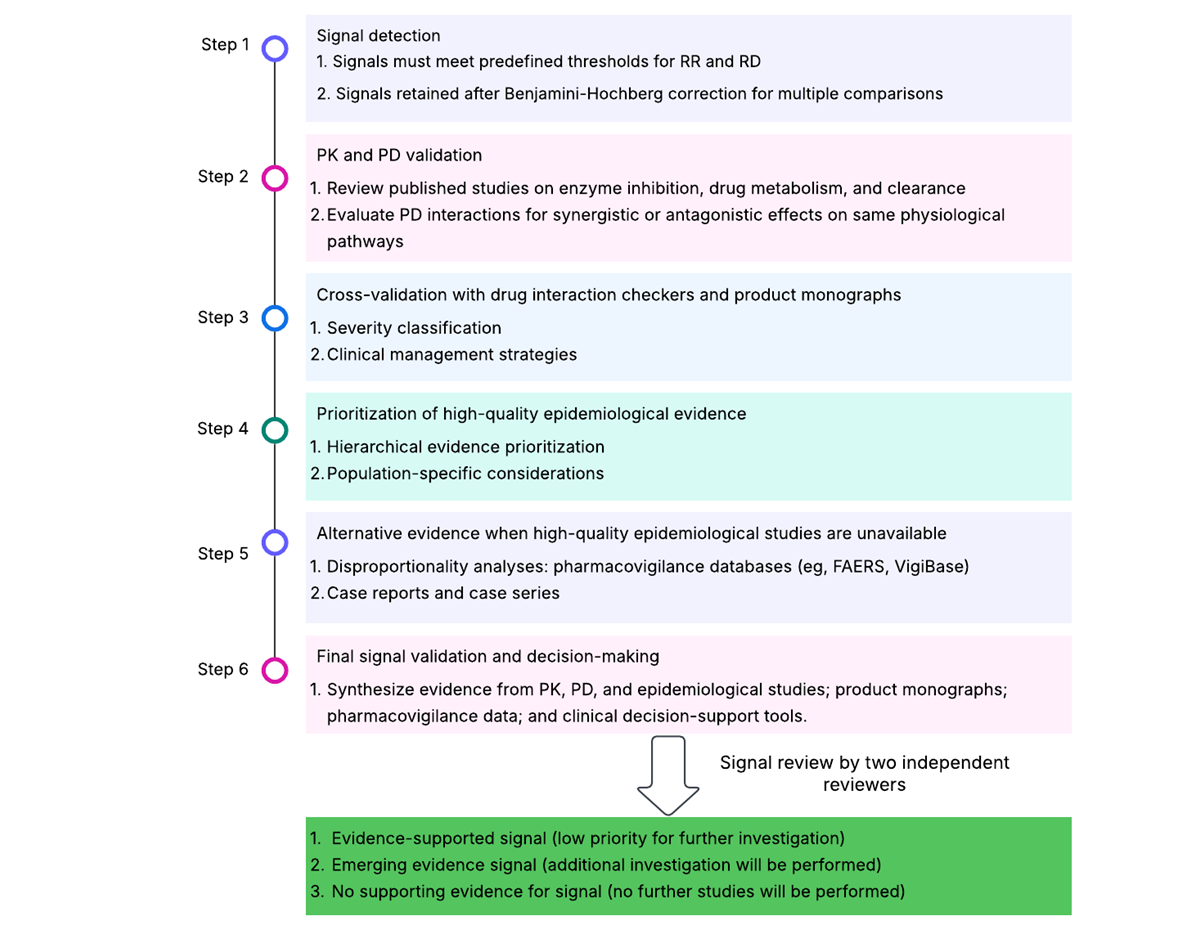
Framework to identify, characterize, and validate signals of harmful DDIs. DDI: drug-drug interaction; PD: pharmacodynamic; PK: pharmacokinetic; RD: risk difference; RR: risk ratio.

Signals identified in the high-throughput computing analysis will be rigorously evaluated for clinical relevance by examining evidence from PK, PD, and epidemiological studies, as well as by using clinical validation tools to prioritize and confirm the most clinically significant interactions as follows (Figure 2):

- Step 1: signal identification. Signals of potentially harmful DDIs identified in the high-throughput analysis must (1) meet predefined thresholds for RRs and RDs, (2) remain statistically significant after the Benjamini-Hochberg correction for multiple comparisons, and (3) be supported in sensitivity and bias analyses.
- Step 2: PK and PD assessment. PK and PD evidence will be evaluated to assess the biological plausibility of the interaction. For PK assessment, published studies on enzyme inhibition, drug metabolism, and clearance will be reviewed. For example, sulfamethoxazole inhibits CYP2C9, which metabolizes warfarin, leading to increased warfarin levels and a higher risk of bleeding [11,45]. For PD assessment, synergistic and antagonistic interactions will be examined. For example, the concurrent use of two antiplatelet agents can increase the risk of bleeding.
- Step 3: cross-validation with drug interaction checkers and product monographs. Signals will be cross-validated using DDI checkers, such as Lexicomp, Micromedex, and Drug Interaction Facts, along with regulatory product monographs (eg, from Health Canada and the US Food and Drug Administration [FDA]). Interaction severity will be classified as major, moderate, or minor based on standardized clinical guidelines. Recommended interventions for major interactions include (1) avoiding coadministration, (2) adjusting doses, and (3) monitoring relevant biomarkers (eg, the International Normalized Ratio [INR] for warfarin interactions).
- Step 4: prioritization of high-quality epidemiological evidence. Evidence from epidemiological studies will be examined and prioritized as randomized controlled trials (RCTs), high-quality cohort studies, and case-control studies. We will also prioritize evidence from studies of vulnerable populations (eg, older adults, patients with chronic kidney disease, and polypharmacy cohorts).
- Step 5: alternative evidence when high-quality epidemiological studies are unavailable. Complementary signal detection methods (eg, disproportionality analyses of pharmacovigilance databases, such as FAERS and VigiBase), case reports, and case series will be reviewed to assess the interaction’s potential clinical significance when high-quality epidemiological evidence is unavailable. For disproportionality analyses, pharmacovigilance databases (eg, FAERS, VigiBase) will be analyzed to determine whether drug coadministration is disproportionately associated with adverse events compared to the administration of a single medication. When large-scale epidemiological data are lacking, case reports and case series may provide supporting evidence.
- Step 6: final signal validation and decision-making. Evidence from PK, PD, and epidemiological studies, pharmacovigilance data, and clinical decision-support tools will be synthesized to assess the credibility of the signal.

Each signal will be independently reviewed by two reviewers using the aforementioned criteria. The reviewers will classify each signal based on the strength and consistency of supporting data as an evidence-supported signal, an emerging evidence signal, or a signal with no supporting evidence. To minimize subjectivity, we will calculate an interrater agreement measure (eg, Cohen κ) to assess the level of concordance between the reviewers and determine whether they reach the same conclusion [46,47]. Any discrepancies will be resolved through discussion and, if needed, adjudicated by a third reviewer.

The criteria for classifying a signal as an evidence-supported signal (regulatory and clinical action may be warranted), an emerging evidence signal (additional investigation will be performed), or a signal having no supporting evidence (no further studies will be performed) are summarized in Table 2. Briefly, a signal will be considered evidence supported if multiple lines of evidence support a causal effect, it will require further study if the existing evidence is conflicting, and it will be dismissed in the absence of supportive evidence.

**Table 2:** Summary table for criteria for signal confirmation, further study, or dismissal.

Category and criteria		Key indicators	Next steps
**Evidence-supported signal^a^ (low priority for further investigation)**			
	Multiple independent sources align	PK^b^/PD^c^ evidence supports a plausible interaction. High-quality epidemiological data (RCTs^d^, cohort studies) confirm increased risk. Product monographs or clinical decision-support tools (eg, Lexicomp) classify it as a major interaction.	Regulatory and clinical action may be warranted, such as labeling changes or guideline updates.
**Emerging evidence signal^a^ (additional investigation to be performed)**			
	Conflicting evidence	PK/PD data suggest a risk, but epidemiological studies do not confirm it. Epidemiological findings are inconsistent or based on low-quality studies (eg, small sample size, case reports only). Product monographs or clinical decision support tools give mixed or uncertain classifications.	A pharmacoepidemiological study should be conducted.
	Limited population-specific data	High-risk populations (patients with chronic kidney disease, older adults, polypharmacy patients) are underrepresented in studies. Current evidence is based on general population data but lacks subgroup analyses.	A pharmacoepidemiological study should be conducted.
	Emerging signal in pharmacovigilance databases	Disproportionality analysis (eg, FAERS, VigiBase) suggests a safety concern but lacks validation in clinical studies. Case reports describe severe but rare adverse events needing further confirmation.	A pharmacoepidemiological study should be conducted.
**No supporting evidence for signal^a^ (no further studies to be performed)**			
	No clear biological or clinical risk	There is a lack of PK/PD plausibility (eg, drugs do not share metabolic pathways or receptor targets). There is no statistically significant association in large epidemiological studies. There are no case reports/case series. Product monographs or clinical decision-support tools classify it as a low-risk (minor) interaction or report no interaction.	No further action is needed.

^a^Signals retained after Benjamini-Hochberg correction for multiple comparisons.

^b^PK: pharmacokinetic.

^c^PD: pharmacodynamic.

^d^RCT: randomized controlled trial.

#### Exploring Harmful DDIs Using Machine Learning

We will explore an alternative strategy for further investigating harmful DDIs using a machine learning algorithm. This complementary approach will integrate key components from our six-step validation framework, including empirical evidence from PK and PD data; findings from epidemiological studies; and clinical validation tools, such as drug interaction checkers (Lexicomp, Micromedex) and product monographs (eg, from Health Canada and the US FDA). We will combine these diverse sources of evidence to train the model, aiming to identify patterns that can help prioritize high-risk DDIs for further investigation. We will also use natural language processing (NLP) algorithms to extract important insights from clinical monographs, published research papers, and other medical literature. This step will help us better understand the context around each signal and provide a richer foundation for analysis. Although still exploratory, this method seeks to refine our ability to distinguish clinically significant interactions from spurious associations, ultimately improving how we prioritize potential risks in clinical practice.

We will first use known, previously validated DDIs identified through our six-step validation process to train the model. All sources of evidence collected—including high-throughput computing, PK and PD data, clinical validation tools (eg, drug interaction checkers and product monographs), epidemiological studies, and pharmacovigilance databases—along with insights from the six-step validation framework, will form a comprehensive training dataset for our machine learning model, enabling it to generate risk scores and rank potential interactions based on predicted clinical significance. To ensure robustness, the model will undergo k-fold cross-validation and bootstrap sampling. We will apply interpretability techniques, such as Shapley additive explanations (SHAP), to identify the most influential factors driving each prediction. By automating signal ranking and prioritization, this machine learning approach will explore new opportunities to enhance the identification of clinically meaningful DDIs, supporting safer prescribing and improved postmarket drug surveillance.

### Ethical Considerations

This research is exempt from Research Ethics Board (REB) review because the data to be used are authorized under Section 45 of Ontario’s Personal Health Information Protection Act (PHIPA), which allows for the use of personal health information for health system evaluation and planning without requiring REB review [48]. The data will be analyzed at ICES [17], an independent, not-for-profit research institute authorized under PHIPA to collect and use personal health information without consent for these authorized purposes. Individual consent from Ontario residents will not be required for this study, as ICES’s legal status under PHIPA authorizes the secondary use of provincial health care records.

All analyses will be performed at ICES using linked, coded (deidentified) data. Researchers will not have access to any identifiable personal information, and strict privacy and security safeguards will be in place to protect confidentiality. No compensation will be provided, as this study will use existing health administrative data and will not involve direct participant recruitment or interaction. This study will not include images, videos, or any identifiable information from individual participants. On completion, the results of this population-based study will be submitted to a peer-reviewed biomedical journal for publication and presented at relevant conferences.

## Results

In a preliminary analysis, we identified approximately 3.8 million older adults who filled prescriptions for over 500 unique medications during the study period. Each drug can serve as drug A or drug B in various combinations, resulting in approximately 200,000 potential drug pairs for analysis. The initial drug pair cohorts had a median of 583 new users per cohort (IQR 237-2130), with a median overlap duration of 57 days (IQR 30-90). For example, in a chronic-chronic drug pair cohort where levothyroxine was drug A and rosuvastatin was drug B, the median overlap duration was 135 days among 128,762 patients, with 73.1% (n=94,036) females and a median age of 66 years. In a chronic-acute drug pair cohort (eg, levothyroxine as drug A and amoxicillin trihydrate as drug B), the median overlap was 10 days among 197,057 patients, with 74.8% (n=147,399) females and a median age of 67 years. In an acute-chronic drug pair cohort (eg, amoxicillin trihydrate as drug A and rosuvastatin as drug B), the median overlap was 10 days among 2702 patients, with 51.6% (n=1394) females and a median age of 66 years. Lastly, in an acute-acute drug pair cohort (eg, amoxicillin trihydrate as drug A and ciprofloxacin as drug B), the median overlap was 10 days among 4220 patients, with 59.4% (n=2507) females and a median age of 70 years. The protocol, finalized on August 30, 2025, outlines the analysis of data from 2002 to 2023. The high-throughput computing analysis is planned for completion by fall 2026, and findings from the analysis will be assessed in 2027 for clinical relevance by integrating PK, PD, and epidemiological evidence, supported by clinical validation tools. The final results will be prepared for dissemination, with manuscript submission planned for December 2028.

## Discussion

### Summary

This study aims to identify harmful DDIs by analyzing Ontario health care databases using high-throughput computing. We will process data from over 3.8 million residents aged 66 years and older, evaluating more than 200,000 medication pairs and examining associations with 74 acute outcomes, including hospitalizations, emergency department visits, and mortality. High-throughput computing will enable us to identify and test thousands of medication combinations. We will compare individuals prescribed medication pairs with those on single medications, using propensity score methods to balance comparison groups on over 400 baseline health characteristics. We will conduct extensive sensitivity and bias analyses to ensure that potential signals are not artifacts of statistical or methodological bias. Additionally, machine learning algorithms will be used to complement the high-throughput computing approach.

High-throughput computing and automated analytical pipelines have proven effective for detecting potential DDIs in large health care datasets in other countries, such as the United States. Previous studies by Bykov et al [14] and Leonard et al [15] have demonstrated that these methods can efficiently confirm known interactions and identify novel signals. However, these approaches have largely been limited to single-object drugs, specific outcomes, or nonrepresentative datasets (derived from a specific subset of the population, such as those with a certain type of insurance, rather than the entire population), which may restrict generalizability. Building on these advances, our study will use high-throughput computing to analyze Ontario’s population-based, linked administrative databases, which capture comprehensive prescription and outcome data across the province’s universal health care system. Using a population-based, new-user cohort design, we will systematically evaluate nearly 200,000 medication pairs among 3.8 million older adults—an effort that would be impractical with traditional methods. This approach will not only fill a critical gap by providing large-scale, real-world evidence in a Canadian context but also enhance generalizability, generate signals that could inform clinical decision-making once validated, and strengthen postmarketing pharmacovigilance efforts for older adults. To the best of our knowledge, this represents the first application of such an approach in Canada, leveraging the strengths of a universal health care setting to further enhance the generalizability and clinical relevance of detected DDI signals.

The cohort study is designed to mimic typical clinical scenarios, for example, an individual who has been taking digoxin for 7 months and is then newly prescribed sulfamethoxazole-trimethoprim to treat a urinary tract infection. In DDI studies, such scenarios are categorized as follows:

- Precipitant triggered: Individuals initiate the object drug first, followed by the precipitant, with follow-up starting on the day the precipitant is initiated.
- Object triggered: Individuals initiate the precipitant first, followed by the object drug, with follow-up starting on the day the object is initiated.

Our approach will be hypothesis free since it does not assume beforehand whether drug A or drug B will function as the object or the precipitant. Analyzing initiation orders separately is highly recommended in traditional DDI studies—if feasible—as it addresses different clinical questions, better controls confounding, and increases the validity of the results [2].

### Strengths of the Study

A key strength of this study is the use of Ontario’s administrative health care databases. These databases include comprehensive, encrypted data on health care visits, hospitalizations, lab results, and prescription records for residents aged 65 years and older [17]. This extensive data coverage enables the capture of a wide range of adverse outcomes, providing a detailed picture of the potential risks associated with specific drug interactions [16].

The application of high-throughput computing and automation will significantly enhance the efficiency and scalability of our analyses [49]. This approach will allow us to simultaneously evaluate numerous drug interactions, which would be impractical using traditional methods. By automating the creation of cohorts and statistical analysis, we can quickly and accurately identify potential DDIs, facilitating more rapid updates to clinical guidelines and regulatory policies [4,50].

### Challenges and Limitations

Despite using advanced statistical techniques to control for confounding, residual confounding may still be present [51]. Factors that are not captured in the administrative data or that are measured with error could bias our results [52]. To address this, we will conduct multiple sensitivity analyses and use negative control outcomes to assess the robustness of our findings [37].

Our study focuses on acute (30-day) outcomes, which will not capture the effect of long-term exposure [53]; however, we will conduct additional analyses to assess longer follow-up times.

The study is designed to identify harmful DDIs with a moderate-to-high risk in magnitude in cohorts of at least 1000 individuals, and therefore, it will have limited statistical power to detect small risk increases.

Our study does not include data on nonprescription (over-the-counter) drugs, drugs prescribed in hospitals, or comprehensive outpatient prescription drug use in children or adults aged under 65 years. These exclusions may limit the generalizability of our findings to a broader population. However, the focus on older adults is justified, given their higher risk of polypharmacy and adverse drug interactions.

Given the large number of comparisons being made, there is a risk of spurious findings due to multiple testing [16]. To mitigate this, we will apply the Benjamini-Hochberg procedure to control the false discovery rate and use E-values to assess the robustness of our findings against unmeasured confounding [31].

### Implications for Clinical Practice and Policy

This study has the potential to significantly improve drug safety, particularly in older adults, by identifying harmful DDIs more efficiently. If replicated in other jurisdictions, the findings could provide clinicians with new insights into the risks associated with specific drug combinations, leading to more informed prescribing practices and enhanced patient monitoring. The evidence generated could also inform regulatory agencies, such as Health Canada and the US FDA, helping refine product label warnings and support regulatory decisions. Ultimately, the findings could contribute to safer medication use on a population level, improving medication safety practices worldwide. Moving forward, we will prioritize and verify high-throughput signals across Ontario and beyond, ensuring the continued refinement and application of this approach to achieve more precise and informed medication safety practices.

### Conclusion

This protocol outlines an innovative approach to detecting harmful DDIs in older adults using high-throughput computing and automation. By leveraging Ontario health care data and using advanced analytical techniques, we aim to enhance the efficiency and comprehensiveness of postmarket drug surveillance. The results of this study, if replicated in other jurisdictions, have the potential to significantly improve medication safety, inform clinical practice, and guide regulatory policies. Ultimately, this research aims to reduce the burden of adverse drug events in older adults and lay the foundation for more precise, data-driven medication safety practices worldwide.

## Appendix

Multimedia Appendix 1 Checklist of recommendations for reporting of observational studies using the RECORD (Reporting of Studies Conducted Using Observational Routinely Collected Health Data) statement.

Multimedia Appendix 2 Study variables and the codes used to ascertain baseline comorbidities (sorted alphabetically).

Multimedia Appendix 3 Process of integrating data from different sources.

Multimedia Appendix 4 Overview of the plan to perform the primary analysis and additional exploratory analysis.

Multimedia Appendix 5 Codes for nonoral drugs.

## Abbreviations

ATT: average treatment-effect-in-the-treated
DDI: drug-drug interaction
FDA: Food and Drug Administration
ICES: Institute for Clinical Evaluative Sciences
IPTW: inverse probability of treatment weighting
NNH: number needed to harm
ODB: Ontario Drug Benefit
PAF: population attributable fraction
PD: pharmacodynamic
PHIPA: Personal Health Information Protection Act
PK: pharmacokinetic
RCT: randomized controlled trial
RD: risk difference
REB: Research Ethics Board
RR: risk ratio
SD: standardized difference
SSRI: serotonin reuptake inhibitor
