# Supplementary material for: High-Throughput Computing to Detect Harmful Drug-Drug Interactions in Older Adults: Protocol for a Population-Based Cohort Study
Source: JMIR Res Protoc. 2025 Oct 10;14:e77224. doi: 10.2196/77224 (PMC12552818; doi:10.2196/77224)
Supplement: Multimedia Appendix 2 [file resprot_v14i1e77224_app2.docx]

**Multimedia Appendix 2: Study variables and the codes used to ascertain baseline comorbidities (sorted alphabetically)**

| **Study variable** | **ICD-10 code** | **CCI code** | **OHIP fee code** | **OHIP diagnosis code** |
| --- | --- | --- | --- | --- |
| Abdominal aortic aneurysm repair/aortic bypass | I713 | 1KA76,1KA80 | R802,R817,R877,R875,E627 |  |
| Achilles tendon rupture | S8600,S8608 |  | R587,R589 |  |
| Acute kidney injury | N170,N171,N172,N178,N179 |  |  |  |
| Alcoholism | E244,E512,F10,G312,G621,G721,I426,K292,K70,K860,T510,X45,X65,Y15,Y573,Z502,Z714,Z721 |  |  | 303 |
| Angina | I200,I209,I2088,I2080,I201,I2382,I2388,I2381 |  |  | 413 |
| Arrhythmia | I4900,I4901,I491,I492,I493,I494,I498,I499,R000,R001,I4890,I480,I4800,I4891,I4802,I481,I4801,I483,I484 |  | G178,G179,G249,G261,G259,Z443,Z431,Z437 |  |
| Arthroscopy |  | 1VG87,1VA80DA,1VA80GZ,1VA83DAXXA,1VA83DAXXP,1VA83HAXXP,1VA87DA,1VA87GB | R686,Z218,E595,E563,R423,R990 |  |
| Asthma | J4590,J4591,J4500,J4510 |  |  |  |
| Atrial fibrillation | I4890,I480,I4800,I4891,I4802,I481,I4801,I483,I484 |  |  |  |
| Bipolar disorder | F300,F301,F302,F308,F309,F310,F311,F312,F313,F314,F315,F316,F317,F318,F319 |  | Q020 | 296 |
| Bone mineral density |  |  | J654,J688,J854,J888,X149,X152,X153,X155,Y654,Y688,Y854,Y888 |  |
| Bowel obstruction | K566 |  |  |  |
| Bradycardia | R001 |  |  |  |
| Bronchoscopy |  |  | E632,E633,E838,E846,Z327,Z330,Z334,Z343,Z348,Z359,Z360 |  |
| Calcium test |  |  | L045,L046 |  |
| Cancer | 971,980,98233,984,985,98613,987,988,989,990,991,993,C153,C180,C19,C20,C220,C250,C3499,C5099,C560,C61,C820,C830,C851,C910,C920,C930,C946,C950,D00,D051,C154,C159,C155,C181,C182,C183,C184,C185,C186,C187,C188,C189,C221,C227,C229,C259,C251,C254,C252,C3410,C3490,C3411,C3430,C3491,C3431,C342,C3400,C3401,C3480,C3409,C3439,C3419,C3481,C5091,C5090,C5041,C5040,C5080,C5081,C5020,C5021,C5051,C5050,C5030,C5010,C5031,C5011,C5000,C561,C569,C821,C822,C829,C831,C833,C837,C838,C852,C857,C859,C911,C914,C915,C919,C921,C929,C927,C925,C928,C931,C951,C959,D0510,D0511,D059,D0591,D0590,D0501 |  |  | 203,204,205,206,207,208,150,154,155,157,162,174,175,183,185 |
| Cardiac catheterization |  | 3IJ30GP,3HZ30GP,2HZ24GPKJ,2HZ24GPKL,2HZ24GPKM,2HZ24GPXJ,2HZ28GPPL,2HZ71GP | G296,G297,G299,G300,G301,G304,G305,G306 |  |
| Cardiac stress test |  | 2HZ08,3IP70 | G315,G174,G111,G112,G319,G582,G583,G584,J604,J606,J607,J608,J609,J611,J612,J613,J667,J807,J808,J809,J804,J811,J812,J813,J867,J666,J866 |  |
| Carotid endarterectomy |  |  | N220,R792 |  |
| Carotid ultrasound |  | 3JE30,3JG30 | J201,J501,J189,J489,J190,J191,J490,J491,J492 |  |
| Cervical cancer screening |  |  | E430,G365,G394,L713,L812 |  |
| Chest X-ray |  |  | X090,X091,X092,X195 |  |
| Cholesterol test |  |  | L117,L055,L056,L156 |  |
| Chronic liver disease | B169,B171,B182,B19,I850,R17,R18,R160,R162,B942,Z225,E831,E830,K70,K713,K714,K715,K717,K721,K729,K73,K74,K753,K754,K758,K759,K76,K77,B179,B181,I859 |  | Z551,Z554 | 571,573,70 |
| Chronic lung disease | I272,I278,I279,J40,J41,J42,J439,J441,J4590,J47,J60,J61,J62,J63,J64,J65,J66,J6799,J68,J701,J703,J704,J708,J709,J82,J841,J920,J941,J949,J953,J961,J969,J984,J988,J989,J99,J449,J440,J448,J439,J4591,J4500,J4510,J848,J849 |  | J889,J689 | 491,492,493,494,496,501,502,515,518,519 |
| Chronic pain | F454,M081,M2550,M2551,M2555,M2556,M2557,M432,M433,M434,M435,M436,M45,M461,M463,M464,M469,M4796,M480,M481,M488,M489,M508,M509,M513,M531,M532,M533,M538,M539,M545,M608,M609,M633,M790,M791,M792,M796,M797,M961,M549,M543,M542,M548,M5416,M546,M544,M5419,M5412,M5417,M4786,M4782,M4792,M4712,M4726,M4797,M4789,M4787,M4722,M511,M512,M519,M518,M510 |  |  |  |
| Clostridium difficile colitis | A047 |  |  |  |
| Colonoscopy |  | 2NM70,1NM87BA,2NM71,1NQ87BA,2NQ71,1NM59,2NQ70,1NQ59BA,1NQ59HA | Z555A,E740A,E741A,E747A,E705A,E749A,Z570A,Z571A,E685A,Z491A,Z492A,Z493A,Z494A,Z495A,Z496A,Z497A,Z498A,Z499A |  |
| Colorectal cancer screening |  |  | G004,L179,L181,Q043,Q152,X112,X113,Z535,Z536,Z555,Z580 |  |
| Colposcopy |  | 2RS70 | Z730,Z731,Z731,Z787 |  |
| COPD (Chronic Obstructive Pulmonary Disease) | J41,J439,J441,J440,J448 |  |  |  |
| Coronary angiogram |  | 3IP10,3IS10 | G297,G509 |  |
| Coronary disease | I214,I229,I240,I2510,Z955,Z958,Z959,R931,T822,I219,I211,I210,I213,I2149,I241,I248,I249,I2519,I252,I255,I259,I2511,I258,I2513,I2514,I250 | 1IJ26,1IJ27,1IJ54,1IJ57,1IJ50,1IJ76 | R741,R742,R743,G298,E646,E651,E652,E654,E655,G262,Z434,Z448 | 410,412 |
| Coronary revascularization |  | 1IJ50,1IJ26,IIJ27,1IJ57,1IJ76,1IJ57GQ,1IJ54GQAZ | R741,R742,R743,E651,E652,E654,E646,G298,Z434,G262 |  |
| CT abdomen |  |  | X126,X409,X410 |  |
| CT extremities |  |  | X127,X412,X413 |  |
| CT head |  | 3AN20,3EA20,3ER20 | X188,X400,X401,X402,X405,X408 |  |
| CT neck |  |  | X124,X403,X404 |  |
| CT pelvis |  |  | X231,X232,X233 |  |
| CT spine |  |  | X128,X415,X416 |  |
| CT thorax |  |  | X125,X406,X407 |  |
| Cutaneous drug reaction | L270,L271,L539,L538,L26,L510,L511,L512,L518,L519 |  |  |  |
| Cystoscopy |  |  | Z606,Z607,Z628,Z632,Z633,Z634 |  |
| Delirium | F050,F051,F058,F059 |  |  |  |
| Dementia | F065,F066,F068,F069,F09,F009,F019,F020,F03,F051,G309,G318,R54,F002,F001,F011,F013,F018,F023,F028,G3088,G3081,G308,G301,G300,G3102,G312,G319,G310 |  |  | 290,331,797 |
| Depression | F063,F064,F320,F321,F322,F323,F328,F329,F330,F331,F332,F333,F334,F338,F339,F341,  F400,F401,F402,F408,F409,F410,F411,F412,F413,F418,F419,F420,F421,F422,F428,F429,F430,F431 |  |  | 311 |
| Diabetic ketoacidosis | E1010,E1012,E1110,E1112,E1310,E1312,E1410,E1412 |  |  |  |
| Digoxin test |  |  | L072,L306 |  |
| Digoxin toxicity | T460,Y520 |  |  |  |
| Echocardiography |  | 3IP30 | G560,G561,G562,G566,G567,G568,G570,G571,G572,G574,G575,G576,G577,G578,G579,G580,G581 |  |
| Electroencephalography |  |  | G414,G415,G416,G417,G418,G540,G542,G544,G545,G546,G554,G555 |  |
| Encephalopathy | F050,F051,F058,F059,R410,R4180,G459 |  |  |  |
| Esophagogastroduodenoscopy |  | 2NK70,2NF71,2NK71,2NA71,2NF70 | Z399,Z400,E696,E702,E690,E795,E770,E692,E698,E703,E799,E695,E797,E798,E629 |  |
| Fall | W00,W01,W02,W03,W04,W05,W06,W07,W08,W09,W10,W11,W12,W13,W14,W15,W16,W17,W18,W19 |  |  |  |
| Fracture | S720,S721,S722,S723,S52,S422,S321,S322,S323,S324,S325,S327,S328 | 1VA73,1VC73,1VA74,1VA53,1VC74,1VA80,1VC03,1VC80,1TV73,1TV74,1TV03 | F014,F022,F023,F025,F026,F028,F030,F032,F033,F046,F024,F027,F031,Z203,F095,F096,F097,Z211 |  |
| GI bleeding (Gastrointestinal bleeding) | I850,I9820,I983,K2210,K2211,K2212,K2214,K2216,K226,K228,K250,K252,K254,K256,K260,K262,K264,K266,K270,K272,K274,K276,K280,K282,K284,K286,K290,K3180,K6380,K920,K921,K5520,K625,K922 |  |  |  |
| Gout | M1000,M1001,M1002,M1003,M1004,M1005,M1006,M1007,M1008,M1009,M1010,M1011,M1012,M1013,M1014,M1015,M1016,M1017,M1018,M1019,M1020,M1021,M1022,M1023,M1024,M1025,M1026,M1027,M1028,M1029,M1030,M1031,M1032,M1033,M1034,M1035,M1036,M1037,M1038,M1039,M1040,M1041,M1042,M1043,M1044,M1045,M1046,M1047,M1048,M1049,M1090,M1091,M1092,M1093,M1094,M1095,M1096,M1097,M1098,M1099 |  |  |  |
| Guillain-Barré syndrome | G610 |  |  |  |
| Head trauma | G913,S061,S065,S066,S060 |  |  | 850 |
| Hearing test |  |  | G153,G154,G440,G441,G442,G443,G448,G450,G451,G452,G525,G526,G529,G530,G533,G815,G816 |  |
| Hearing loss | H919,H918,H905 |  | Z914,E346 | 381,382 |
| Heart failure | I500,I501,I509,I255,J81 | 1HP53,1HP55,1HZ53GRFR,1HZ53LAFR,1HZ53SYFR | R701,R702,Z429 | 428 |
| Hepatitis B | B169,B180,B181 |  |  |  |
| Herpes simplex | B009,A600,B001,B005,B002,B008,B004 |  |  | 54 |
| Holter monitoring |  | 2HZ24JAKH | G311,G320,G647,G648,G649,G650,G651,G652,G653,G654,G655,G656,G657,G658,GG59,G660,G661,G682,G683,G684,G685,G686,G687,G688,G689,G690,G692 |  |
| Hypercalcemia | E835 |  |  |  |
| Hyperglycemia | R739,E1010,E1012,E1110,E1112,E1310,E1312,E1410,E1412,E1100,E1101,E1300,E1301,E1400,E1401,E11,E13,E14 |  |  |  |
| Hyperkalemia | E875 |  |  |  |
| Hyperosmolar nonketotic coma | E1100,E1101,E1300,E1301, E1400,E1401 |  |  |  |
| Hypoglycemia | E15,E160,E161,E162,E1063,E1163,E1363,E1463 |  |  |  |
| Hypokalemia | E876 |  |  |  |
| Hypomagnesemia | E834 |  |  |  |
| Hyponatremia | E871 |  |  |  |
| Hypotension | I959,I951,I952,I958,I950 |  |  |  |
| Hypothyroidism | E030,E031,E032,E033,E034,E035,E038,E039,E890 |  |  | 243,244 |
| ICU admissions | G557,G558,G559,G400,G401,G402,G405,G406,G407 | 1GZ31CAND,1GZCRND,1GZ31GPND |  |  |
| GI bleeding | I600,I601,I602,I603,I604,I605,I606,I607,I609,I619,I620,I629,I611,I618,I615,I610,I614,I613,I616 |  |  |  |
| Influenza vaccination |  |  | G590,G591 |  |
| Kidney stones | N200,N210,N23,N132,N136,N201,N202,N289,N2888,N209,N211 |  | Z630,E773,Z629,Z623,J046,E759,E772 |  |
| Laparoscopy |  | 2OT70 | E792,E860,E862,E863,S310,S315,S400,S653,S746,S805,S806,S807,S808,S810,Z718 |  |
| Lithium toxicity | T438,T439,T568,T569,T435,Y495 |  |  |  |
| Liver disease toxicity | K729,K711,K720,K759,K716,K719,K746,K762,K769,Z944 |  |  |  |
| MACE (Major Adverse Cardiovascular Events) | H341,I630,I631,I633,I634,I635,I638,I639,I64,I632,I500,I509,I501,I214,I219,I211,I210,I213,I2149,I212,I229,I220,I221 |  |  |  |
| Major hemorrhage | I600,I601,I602,I603,I604,I605,I606,I607,I609,I619,I620,I850,I9820,I983,K2210,K2211,K2212,K2214,K2216,K226,K228,K250,K252,K254,K256,K260,K262,K264,K266,K270,K272,K274,K276,K280,K282,K284,K286,K290,K3180,K6380,K920,K921,K5520,K625,K922,R58,M2509,M2501,M2502,M2503,M2504,M2505,M2506,M2507,M2508,M2500,M1229,M1221,M1222,M1223,M1224,M1225,M1226,M1227,M1228,M1220,I611,I618,I615,I610,I614,I613,I616,I629 |  |  |  |
| Major surgery |  |  | S073,S074,S075,S080,S081,S082,S083,S084,S085,S086,S087,S088,S089,S090,S091,S092,S093,S095,S096,S097,S098,S099,S100,S102,S117,S120,S121,S122,S123,S124,S125,S128,S129,S131,S132,S133,S134,S135,S137,S138,S139,S140,S149,S150,S151,S154,S155,S156,S157,S158,S159,S160,S161,S162,S164,S165,S166,S167,S168,S169,S170,S171,S172,S173,S174,S175,S176,S177,S178,S179,S180,S181,S182,S183,S184,S185,S187,S188,S189,S191,S192,S193,S194,S195,S196,S197,S199,S201,S202,S203,S204,S205,S206,S213,S214,S215,S216,S217,S218,R700,R701,R702,R703,R704,R705,R708,R712,R713,R714,R715,R716,R717,R718,R720,R721,R722,R723,R724,R725,R726,R727,R728,R729,R730,R733,R734,R735,R736,R737,R738,R741,R742,R743,R746,R747,R748,R749,R750,R754,R755,R756,R757,R758,R759,R760,R761,R762,R763,R764,R765,R768,R769,R770,R771,R772,R773,R774,R780,R783,R784,R785,R786,R787,R788,R789,R790,R791,R792,R794,R795,R796,R797,R798,R799,R800,R801,R802,R803,R804,R805,R806,R807,R808,R809,R810,R811,R812,R813,R814,R815,R816,R817,R830,R831,R832,R833,R855,R856,R857,R858,R859,R860,R861,R862,R863,R864,R865,R875,R876,R877,R880,R881,R882,R883,R920,R921,R922,R923,R924,R925,R926,R927,R928,R929,R930,R932,R933,R934,R935,R936,R937,R622,R624,R625,R626,R630,R631,F004,F005,F006,F007,F008,F009,F010,F011,F012,F013,F014,F015,F016,F017,F018,F019,F020,F021,F022,F023,F024,F025,F026,F027,F028,F029,F030,F031,F032,F033,F034,F035,F036,F037,F038,F039,F040,F041,F042,F043,F044,F045,F046,F047,F048,F049,F050,F051,F052,F053,F054,F055,F056,F057,F058,F059,F060,F061,F062,F063,F064,F065,F066,F067,F068,F070,F071,F072,F074,F075,F076,F077,F078,F079,F080,F081,F082,F083,F084,F085,F087,F094,F095,F096,F097,F098,F099,F100,F101,F102,F103,F104,F105,F107,F108,F110,F115,F118,F119,F120,F121,F122,F123,F124,F125,F130,F131,F134,F135,F136,F137,F138,F139,F140,F142,F143,F144,F146,F150,F200,F201,D001,D003,D004,D006,D007,D008,D009,D010,D011,D012,D014,D015,D016,D017,D023,D025,D026,D027,D028,D029,D030,D031,D032,D033,D034,D035,D036,D038,D039,D040,D041,D042,D043,D046,D047,D052,D059,D060,D061,D062,D063,Z197,Z198,Z199,Z200,Z201,Z202,Z203,Z204,Z205,Z206,Z207,Z208,Z209,Z210,Z211,Z212,Z213,Z214,Z215,Z216,Z217,Z218,Z222,Z223,Z224,Z225,Z226,Z227,Z228,Z229,Z230,Z231,Z232,Z233,Z234,Z235,Z236,Z237,Z238,Z239,Z240,Z241,Z242,Z243,Z244,Z245,Z246,Z247,Z248,Z249,Z250,Z251,Z252,Z253,Z254,Z255,Z256,Z257,Z258,Z259,Z260,Z261,Z262,Z263,Z264,Z265,Z266,Z267,Z268,Z269,Z270,Z271,S002,S003,S004,S005,S006,S007,S010,S011,S012,S013,S014,S015,S018,S019,S020,S021,S023,S024,S025,S028,S030,S031,S032,S033,S034,S035,S036,S042,S043,S044,S045,S046,S047,S049,S050,S057,S058,S059,S061,S062,S063,S065,S066,S067,S068,S069,S103,S104,S113,S114,S115,S116,S118,S119,S208,S209,S222,S223,S225,S226,S227,S228,S229,S231,S233,S234,S236,S237,S241,S242,S243,S246,S247,S248,S249,S251,S253,S256,S257,S258,S259,S260,S265,S266,S267,S268,S269,S270,S271,S272,S273,S274,S275,S276,S278,S280,S281,S282,S283,S284,S285,S287,S291,S292,S293,S294,S295,S297,S298,S299,S300,S301,S302,S303,S304,S305,S306,S307,S308,S309,S310,S311,S312,S313,S314,S315,S316,S317,S318,S319,S320,S321,S322,S323,S325,S326,S328,S329,S330,S332,S333,S334,S335,S336,S337,S338,S339,S340,S342,S343,S344,S345,S346,S347,S348,S349,S355,S372,S400,S401,S402,S403,S404,S405,S406,S407,S408,S409,S410,S411,S412,S413,S415,S416,S417,S418,S420,S421,S422,S423,S424,S426,S427,S428,S429,S430,S431,S432,S433,S434,S435,S436,S437,S438,S440,S441,S442,S443,S444,S445,S446,S447,S448,S449,S450,S451,S452,S453,S454,S455,S456,S457,S458,S459,S460,S461,S462,S463,S465,S466,S467,S468,S470,S471,S476,S477,S478,S479,S480,S481,S482,S483,S484,S485,S487,S488,S489,S490,S491,S502,S512,S513,S518,S519,S520,S521,S522,S523,S524,S525,S530,S531,S532,S533,S534,S535,S536,S537,S538,S539,S540,S541,S542,S543,S544,S545,S546,S547,S548,S549,S550,S551,S552,S553,S554,S555,S556,S557,S558,S559,S560,S561,S562,S563,S564,S566,S567,S568,S569,S570,S571,S572,S573,S574,S575,S576,S577,S578,S579,S580,S581,S588,S589,S590,S591,S592,S593,S595,S596,S597,S598,S599,S600,S601,S602,S606,S611,S616,S618,S619,S623,S625,S626,S630,S631,S636,S640,S641,S642,S643,S644,S645,S646,S647,S648,S649,S650,S651,S652,S653,S654,S655,S656,S700,S701,S702,S703,S704,S705,S706,S707,S708,S709,S710,S711,S712,S713,S714,S715,S716,S717,S718,S719,S720,S721,S722,S723,S724,S725,S726,S727,S728,S729,S730,S731,S732,S733,S734,S735,S736,S737,S738,S739,S740,S741,S742,S743,S744,S745,S746,S747,S748,S749,S750,S751,S752,S753,S754,S755,S756,S757,S758,S759,S760,S761,S762,S763,S764,S765,S766,S767,S768,S769,S770,S771,S772,S773,S774,S775,S776,S777,S778,S779,S780,S781,S782,S783,S784,S785,S786,S787,S788,S789,S790,S791,S792,S793,S795,S796,S797,S798,S799,S800,S805,S806,S807,S808,S810,S811,S812,S813,S815,S816,S900,R107,R108,R109,R110,R111,R112,R113,R114,R115,R116,R117,R118,R119,R120,R121,R122,R123,R124,R143,R144,R145,R146,R147,R148,R149,R150,R151,R152,R153,R154,R155,R156,R181,R182,R191,R192,R193,R194,R195,R196,R197,R198,R199,R200,R201,R202,R203,R204,R205,R206,R207,R208,R209,R210,R211,R212,R213,R214,R215,R216,R217,R218,R219,R220,R221,R222,R223,R224,R225,R226,R227,R228,R229,R230,R231,R232,R233,R234,R235,R236,R237,R238,R239,R240,R241,R242,R243,R244,R245,R246,R247,R248,R249,R250,R251,R252,R253,R254,R255,R256,R257,R258,R259,R260,R261,R262,R263,R264,R265,R266,R267,R268,R269,R270,R271,R272,R273,R274,R275,R276,R277,R278,R279,R280,R281,R282,R283,R284,R285,R286,R287,R288,R289,R290,R291,R292,R293,R294,R295,R296,R297,R298,R299,R301,R302,R303,R304,R305,R306,R307,R308,R309,R310,R311,R312,R313,R314,R315,R316,R317,R318,R319,R320,R321,R322,R323,R324,R325,R326,R327,R328,R329,R330,R331,R332,R333,R334,R335,R336,R337,R338,R339,R340,R341,R342,R343,R344,R345,R346,R347,R348,R349,R350,R351,R352,R353,R354,R355,R356,R357,R358,R359,R360,R361,R362,R363,R364,R365,R366,R367,R368,R369,R370,R371,R372,R373,R374,R376,R377,R378,R379,R380,R381,R382,R383,R384,R385,R386,R387,R388,R389,R390,R391,R392,R393,R394,R395,R396,R397,R398,R399,R400,R401,R402,R403,R404,R405,R406,R407,R408,R409,R410,R411,R412,R413,R414,R415,R416,R417,R418,R419,R420,R421,R422,R423,R424,R425,R426,R427,R428,R429,R430,R431,R432,R433,R434,R435,R436,R437,R438,R439,R440,R441,R442,R443,R444,R445,R446,R447,R448,R449,R450,R451,R452,R453,R454,R455,R456,R457,R458,R459,R460,R461,R462,R463,R464,R465,R466,R467,R468,R469,R470,R471,R472,R473,R474,R475,R476,R477,R478,R479,R480,R481,R482,R483,R484,R485,R486,R487,R488,R489,R490,R491,R492,R493,R494,R495,R496,R497,R498,R499,R500,R501,R502,R503,R504,R505,R506,R507,R508,R509,R510,R511,R512,R513,R514,R515,R516,R517,R518,R519,R520,R521,R522,R523,R524,R525,R526,R527,R528,R529,R530,R531,R532,R533,R534,R535,R536,R537,R538,R539,R540,R541,R542,R543,R544,R545,R546,R547,R548,R549,R550,R551,R552,R553,R554,R555,R556,R557,R558,R559,R560,R561,R562,R563,R564,R565,R566,R567,R568,R569,R570,R571,R572,R573,R574,R575,R576,R577,R578,R579,R580,R581,R582,R583,R584,R585,R586,R587,R588,R589,R590,R591,R592,R593,R594,R595,R596,R597,R598,R599,R600,R601,R602,R603,R604,R605,R606,R607,R608,R609,R610,R611,R612,R613,R614,R615,R616,R617,R618,R619,R620,R623,R621,R627,R628,R629,R632,R633,R634,R635,R636,R637,R638,R639,R640,R641,R642,R643,R644,R645,R646,R647,R648,R649,R650,R651,R652,R653,R654,R655,R656,R657,R658,R659,R675,R676,R677,R678,R679,R680,R681,R682,R683,R684,R685,R686,R687,R688,R689,R690,R691,R692,R693,R694,R695,R696,R697,R698,R706,R709,R710,R711,R751,R752,R753,R775,R776,R778,R781,R818,R819,R820,R821,R822,R823,R824,R825,R826,R827,R828,R829,R834,R835,R836,R837,R838,R839,R840,R841,R842,R843,R844,R846,R848,R849,R850,R851,R852,R853,R854,R866,R867,R868,R869,R870,R872,R873,R874,R878,R879,R885,R905,R907,R910,R911,R912,R913,R914,R915,R916,R940,R941,R942,R943,R944,R945,R946,R950,R951,R952,R953,R954,R956,R957,R958,R959,R960,R961,R962,R963,R964,R965,R966,R967,R968,R969,R970,R971,R972,R973,R974,R975,R976,R977,R978,R979,R990,R991,R993,R999,F000,F001,F002,F218,F627,Z219,Z220,Z221,Z273,Z279,Z280,Z281,Z290,Z291,Z296,Z297,Z298,Z299,Z301,Z302,Z303,Z304,Z305,Z306,Z308,Z309,Z310,Z311,Z312,Z313,Z314,Z315,Z316,Z317,Z318,Z319,Z320,Z321,Z322,Z323,Z324,Z325,Z326,Z327,Z328,Z329,Z330,Z331,Z332,Z333,Z334,Z335,Z336,Z337,Z338,Z339,Z340,Z341,Z342,Z343,Z344,Z345,Z346,Z347,Z348,Z349,Z350,Z351,Z353,Z354,Z355,Z356,Z357,Z358,Z359,Z399,Z400,Z401,Z402,Z408,Z409,Z410,Z411,Z412,Z413,Z414,Z415,Z422,Z423,Z424,Z425,Z426,Z427,Z428,Z429,Z430,Z431,Z432,Z433,Z434,Z435,Z436,Z437,Z438,Z439,Z440,Z441,Z442,Z443,Z444,Z445,Z446,Z447,Z448,Z449,Z450,Z451,Z452,Z453,Z454,Z455,Z456,Z457,Z458,Z459,Z460,Z461,Z462,Z463,Z464,Z465,Z466,Z470,Z475,Z477,Z478,Z480,Z496,Z497,Z498,Z499,Z512,Z513,Z514,Z515,Z520,Z523,Z524,Z525,Z526,Z527,Z528,Z529,Z530,Z531,Z532,Z533,Z534,Z535,Z536,Z537,Z538,Z539,Z540,Z541,Z542,Z543,Z544,Z545,Z546,Z547,Z548,Z549,Z550,Z551,Z552,Z553,Z554,Z555,Z556,Z557,Z558,Z559,Z560,Z561,Z562,Z563,Z564,Z565,Z566,Z567,Z568,Z569,Z570,Z571,Z572,Z573,Z574,Z575,Z576,Z577,Z578,Z579,Z580,Z581,Z582,Z583,Z584,Z585,Z586,Z587,Z590,Z591,Z592,Z593,Z594,Z595,Z596,Z597,Z600,Z601,Z602,Z603,Z604,Z605,Z606,Z607,Z608,Z609,Z610,Z611,Z612,Z615,Z616,Z617,Z618,Z619,Z620,Z621,Z622,Z623,Z624,Z625,Z626,Z627,Z628,Z629,Z630,Z631,Z632,Z633,Z634,Z635,Z636,Z637,Z638,Z640,Z662,Z700,Z701,Z702,Z703,Z704,Z705,Z706,Z707,Z708,Z709,Z710,Z711,Z712,Z713,Z714,Z715,Z716,Z717,Z718,Z719,Z720,Z721,Z722,Z723,Z724,Z725,Z726,Z727,Z728,Z734,Z735,Z736,Z737,Z738,Z739,Z740,Z741,Z742,Z743,Z744,Z745,Z746,Z747,Z748,Z749,Z750,Z751,Z752,Z753,Z754,Z755,Z756,Z757,Z758,Z759,Z760,Z761,Z762,Z763,Z764,Z765,Z766,Z767,Z768,Z769,Z771,Z772,Z773,Z774,Z775,Z776,Z777,Z778,Z779,Z780,Z781,Z782,Z783,Z784,Z785,Z787,Z788,Z800,Z801,Z802,Z803,Z804,Z805,Z806,Z807,Z808,Z809,Z810,Z811,Z812,Z813,Z814,Z815,Z816,Z817,Z818,Z819,Z820,Z821,Z823,Z824,Z825,Z826,Z827,Z869,Z870,Z873,Z941,Z942,Z943,Z944 |  |
| Malnutrition | E40,E41,E42,E43,E44,E45,E46 |  |  |  |
| Mammography |  |  | X172,X178,X184,X185,X201 |  |
| Myocardial infarction | I214,I219,I211,I210,I213,I2149,I212,I229,I220,I221,I228 |  |  |  |
| Obesity | E660,E661,E662,E668,E669 |  |  | 278 |
| Obstructive hypertrophic cardiomyopathy | I421 |  |  |  |
| Osteoarthritis | M159,M150,M169,M160,M161,M165,M167,M179,M170,M171,M173,M189,M199,M190,M198,M191,M4796,M4786,M4782,M4792,M4712,M4726,M4797,M4789,M4787,M4722 |  |  |  |
| Pancreatitis | B252,B263,K860,K861,K859,K851,K858,K852,K850,K853 |  |  |  |
| Pancytopenia | D611,D612,D613,D618,D619,D700,D708,D728,D696,D6938 |  |  |  |
| Parkinson's disease | F023,G20 |  |  |  |
| Peripheral vascular disease | I700,I702,I708,I709,I731,I738,I739,K551 | 1KA76,1KA50,1KE76,1KG26,1KG50,1KG57,1KG76MI,1KG87,1IA87LA,1IB87LA,1IC87LA,1ID87,1KA87LA,1KE57 |  |  |
| Pneumonia | J129,J121,J128,J123,J13,J14,J159,J152,J151,J158,J150,J156,J155,J154,J16,J170,J172,J178,J173,J189,J180,J181,J188,J101,J100,J108,J111,J118,J110 |  |  |  |
| Prostate-specific antigen test |  |  | Q005,Q118,Q119,Q120,Q121,Q122,Q123,Q133 |  |
| Pulmonary function test |  |  | J301,J303,J304,J305,J306,J307,J308,J309,J310,J311,J313,J315,J316,J317,J318,J319,J320,J322,J323,J324,J327,J328,J330,J331,J332,J333,J334,J335,J340,J341,E450,E451 |  |
| Renal biopsy |  | 2PC71BA,2PC71DA,2PC71HA,2PC71LA,2PE71BA,2PE71DA,2PE71HA,2PE71LA | Z601,E820 |  |
| Retinal detachment | H330,H331,H332,H333,H334,H335 |  | E152,E142,E148,E936 |  |
| Rhabdomyolysis | M628,T796 |  |  |  |
| Rheumatoid arthritis | M051,M069,M064,M068 |  |  |  |
| Schizophrenia | F060,F062,F105,F107,F115,F117,F125,F127,F135,F137,F145,F147,F155,F157,F165,F167,F175,F177  ,F185,F187,F195,F197,F200,F201,F202,F203,F204,F205,F206,F208,F209,F220,F228,F229,F230,F231,  F232,F233,F238,F239,F24,F250,F251,F252,F258,F259,F28,F29 |  | Q021 | 291,292,295,297,298 |
| Seizures | R5680,R5688,G4090,G4060,G4020,G4010,G4030,G409,G410,G419 |  |  |  |
| Self-harm | X60,X61,X62,X63,X64,X65,X66,X67,X68,X69,X70,X71,X72,X73,X74,X75,X76,X77,X78,X79,X80,X81,X82,X83,X84 |  |  |  |
| Sepsis | A021,A392,A394,A400,A401,A402,A408,A409,A410,A411,A403,A412,A414,A4159,A413,A4150,A4151,A4152,A4158,A4180,A4188,A427,A419 |  |  |  |
| Severe respiratory depression |  |  | G405,G557,G558,G559,G406,G407 |  |
| Stroke | I602,I603,I604,I605,I606,I607,I609,I619,G450,G451,G452,G453,G458,G459,H340,I611,I618,I615,I610,I614,I613,I616 |  |  |  |
| Syncope | R55 |  |  |  |
| Thyrotoxicosis | E050,E051,E052,E053,E054,E055,E058,E059 |  |  |  |
| Transurethral resection of the prostate |  | 1QT59BAAD,1QT59BAAG,1QT59BAAW,1QT59BAAZ,1QT59BACG,1QT59BAGX,1QT87BA,1QT87BAAG,1QT87BAAK | S655 |  |
| TSH test (Thyroid-stimulating hormone test) |  |  | G016,G399,L341 |  |
| Urinary tract infection | N10,N111,N12,N136,N151,N159,N160,N300,N308,N309,N340,N390,N410,N411,N412,N413,N431,N45,T835,N119 |  |  |  |
| Urinary incontinence | N393,N394,R32 |  |  |  |
| Urine culture |  |  | L253,L254,L255,L633,L634,L641,G009,G010 |  |
| Venous thromboembolism | I801,I802,I803,I822,I828,I829,O871,O878,O879,I269,O882,I260 |  | J198,J498,J493,J202,J502,J659,J660,J859,J860,X406,X407,X125 | 451,671,415,677 |
| Ventricular tachycardia | I4900,I4901,I470,I472,I460,I469 |  |  |  |
