# Supplementary figures and images for: High-Throughput Computing to Detect Harmful Drug-Drug Interactions in Older Adults: Protocol for a Population-Based Cohort Study
Source: JMIR Res Protoc. 2025 Oct 10;14:e77224. doi: 10.2196/77224 (PMC12552818; doi:10.2196/77224)

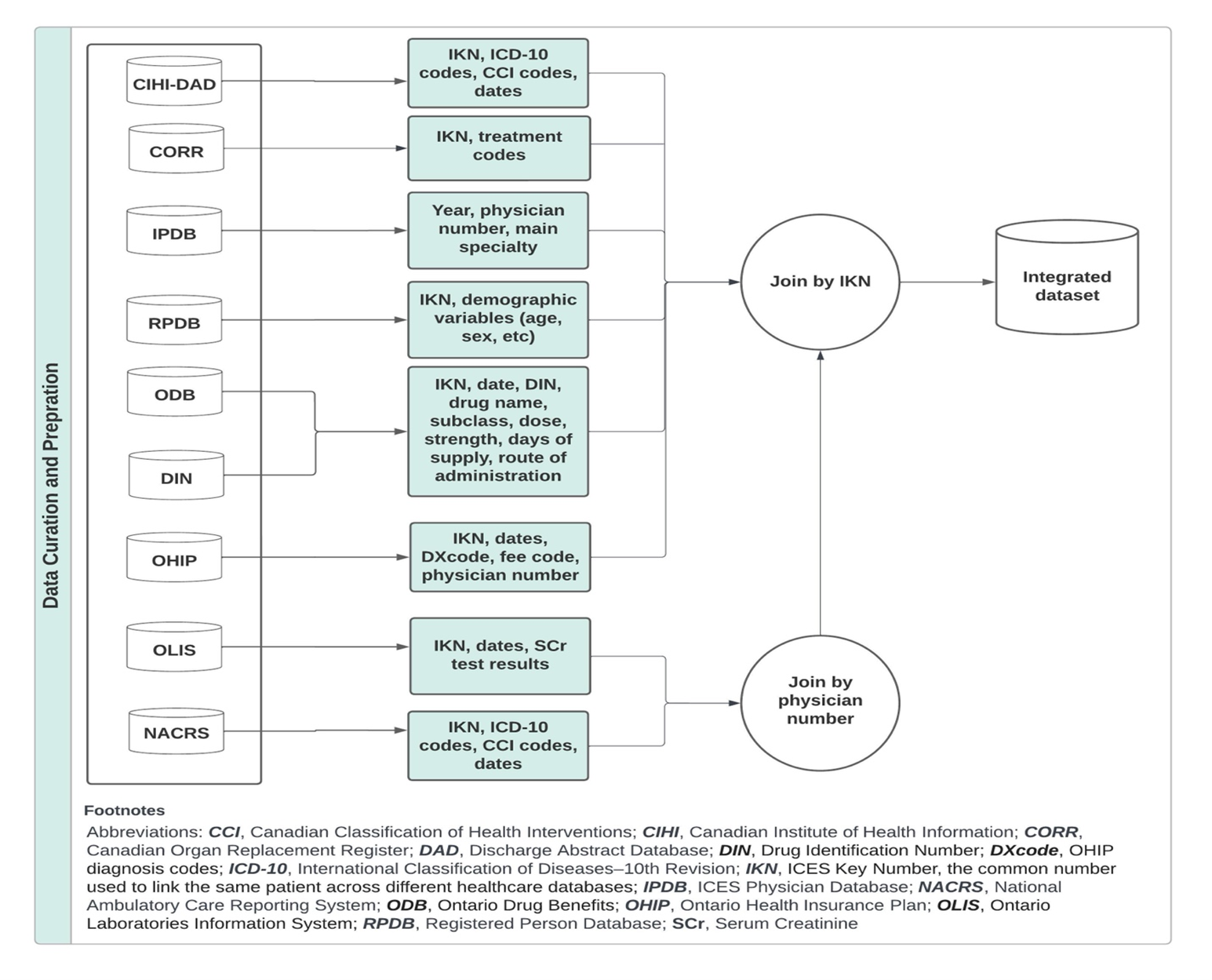

Supplement: Multimedia Appendix 3 [file resprot_v14i1e77224_app3.png]

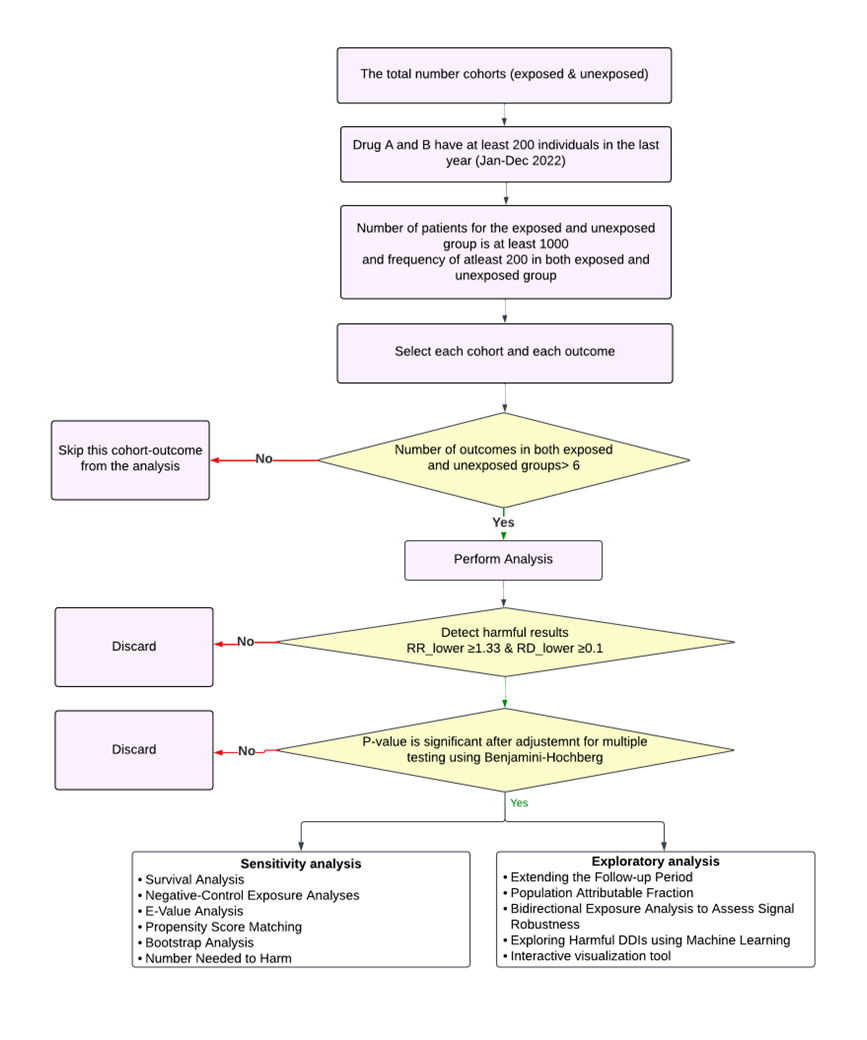

Supplement: Multimedia Appendix 4 [file resprot_v14i1e77224_app4.png]
