# Supplementary material for: High-Throughput Computing to Detect Harmful Drug-Drug Interactions in Older Adults: Protocol for a Population-Based Cohort Study
Source: JMIR Res Protoc. 2025 Oct 10;14:e77224. doi: 10.2196/77224 (PMC12552818; doi:10.2196/77224)
Supplement: Multimedia Appendix 5 [file resprot_v14i1e77224_app5.docx]

**Multimedia Appendix 5. All prescriptions of non-oral drugs where the route of administration is not one of the following will be removed from the study**

TAB, FC TAB, TABLET, TAB DR, CAP, CAPLT, CAP SR, ENT TAB, TAB ENT, TAB ENT SR, CAPLT ENT, TAB CHEW, IR CAP, TAB SR, TAB ER, ER TAB, CAPSULE, CAP ER, ER CAP, Chew Tab, TAB SL, TAB FC, SL TAB, GEL CAP, TAB EFF, IR TAB, TAB CR, CR TAB, TAB SUSP, TAB O/DIS, SR CAP, EC TAB, CAP ENT, CAP CD, CD CAP, DR CAP, CAP/TAB, SPRINKLE CAP, DR TAB, CAP DR, SOFTGEL CAP, INH CAP, INHAL CAP, CR Cap, TAB DR ER, SR TAB, TABLET ORALLY DIS, or TAB RD
